# Can surgical site infections be controlled through microbiological surveillance? A three-year laboratory-based surveillance at an orthopaedic unit, retrospective observatory study

**DOI:** 10.1007/s00264-019-04298-x

**Published:** 2019-01-24

**Authors:** Iwona Pawłowska, Grzegorz Ziółkowski, Jadwiga Wójkowska-Mach, Tomasz Bielecki

**Affiliations:** 1 Division of Microbiology and Epidemiology, St. Barbara Specialised Regional Hospital No. 5, Sosnowiec, Poland; 2 Sosnowiec Medical College, Sosnowiec, Poland; 3 Department of Microbiology, Faculty of Medicine, Jagiellonian University Medical College, 18 Czysta St., 31-121 Kraków, Poland; 4 Medical University of Silesia, Katowice, Poland

**Keywords:** Orthopaedics, Laboratory-based surveillance, Surgical site infections, Multidrug-resistant microorganisms, Cumulative antibiogram

## Abstract

**Objective:**

The aims of the study were to analyse the surgical site infections (SSIs) in patients operated at an orthopaedic ward and to describe the drug-resistance of the aetiology of those infections. Also, analyse the possibility of SSI control through microbiological surveillance. Additionally, we have studied the information inferred by aggregating cumulative antibiograms for the SSIs of the studied orthopaedic unit.

**Design:**

Cross-sectional studies carried out in 2013–2015.

**Setting and patients:**

Orthopaedic and Trauma Surgery Unit in Sosnowiec, Poland; 5995 patients, 5239 operations.

**Methods:**

Retrospective laboratory-based data collection study of surgical site infections.

**Results:**

SSI incidence rate was 6.6%, in the implantations—hip prosthesis 5.8% and knee prosthesis 5.4%, about 6 times higher compared with European HAI-Net. SSIs were usually caused by Gram-positive bacteria (56%). The prevalence of MDR microorganisms was 22.6%, and mainly concerned the Gram-negative bacilli: 97.6% of *Acinetobacter baumannii* and 50.0% of *Klebsiella pneumoniae* were multidrug-resistant. On the basis of what the Formula for Rational Empiric Antimicrobial Therapy analysis has shown, the use of amikacin, imipenem and ciprofloxacin has been recommended as the most efficient in the empirical therapy of SSIs.

**Conclusions:**

The infection control was a significant problem at the studied orthopaedic unit, as evidenced by the SSI incidence rate significantly higher than expected. We suggest implementing the infection control and prevention based on evidence-based medicine, and a unit-based surveillance. A cumulative unit-based antibiogram reflects the drug-susceptibility pattern for the strains from the infections acquired at the unit.

## Introduction

Surveillance is a systematic collection, analysis and interpretation of health data. It is essential for the planning, implementation and evaluation of public health practice, especially when hospital-acquired infections (HAIs) and antimicrobial resistance are concerned [1].

The European Centre for Disease Control and Prevention (ECDC) recommends an active, targeted surveillance of certain types of HAIs, including surgical site infections (SSIs). The incidence of SSIs in surgical operations is estimated at 1.4–20%, depending on the procedure implemented: it amounts to 1–3% for a primary arthroplasty, but is significantly higher for a revision arthroplasty. The causative pathogens depend on the type of surgery; the main etiological agents are Gram-positive cocci, especially Staphylococcus spp.: *Staphylococcus aureus* and coagulase-negative staphylococci, in the endoarthroplasty, their share amounting to 70%, in orthopaedic trauma–related SSI about 35% [2–5].

Numerous patient- and procedure-related factors influence the risk of SSI. Potential patient-related factors include malnutrition (serum albumin concentration), higher age, coexisting infection and diabetes. External factors include the type and duration of the operation, the quality of pre-operative skin preparation, adequacy and timing of antimicrobial prophylaxis, the insertion of foreign materials or implants, hair removal, insufficient environmental hygiene or hand hygiene, and a bad work organisation at the operating theatre. The risk is also elevated by a prolonged pre-operative hospitalisation [6, 7].

Consequences of SSIs may be grave for both the patients and the hospital. On average, SSIs extend the length of hospital stay by two weeks, double the number of re-hospitalizations and triple the cost of treatment—in comparison with the patients with no SSIs; in Poland, SSIs prolong the hospitalisation period more than three times [7, 8].

The incidence of infections may be higher due to a significant rise in the number of implantations performed, and in the age of the patients [9, 10].

The aims of the study were to analyse the epidemiology and microbiology of the SSIs in patients operated at an orthopaedic ward and to describe the drug resistance of the aetiology of those infections. Additionally, we have studied the information inferred by aggregating cumulative antibiograms for the SSIs of the studied unit.

## Materials and methods

The laboratory-based study was conducted between 2013 and 2015 among 5995 patients of the Department and Clinic of Orthopaedic and Trauma Surgery, St. Barbara Specialised Regional Hospital No. 5, Sosnowiec. The hospital has the largest emergency department in the region of Silesia in southern Poland. The 38-bed department (including 6 beds at the paediatric subunit) used for the research was a teaching unit where students and residents are being trained. Emergencies constituted 5% of all admissions to the unit. Five thousand two hundred thirty-nine operations were performed in the studied period, and the average length of hospitalisation was 8.8 days.

An independent microbiological laboratory working at the hospital conducts some 2400 investigations of the clinical materials for the unit per year. The infection control team was composed of three epidemiological nurses, a doctor specialised in contagious diseases and a microbiologist specialised in diagnosis. Our experiences from the previous years have already been partly discussed, but those discussions concerned different types of infections and patient populations [11, 12].

Three hundred forty-five SSI cases have been detected. The aetiological factor could not be isolated in ten of them. Four hundred two strains of the species considered as aetiological factors of SSIs have been analysed.

In compliance with the International Classification of Diseases, Ninth Revision, Clinical Modification (ICD-9-CM), and with the methodology of the National Healthcare Safety Network (NHSN), the operations performed have been divided into: hip arthroplasties (HPROs), knee arthroplasties (KPROs), other prostheses (OPROs) and other musculoskeletal surgery (OMSs). Cephazolin was applied in the pre-operative antibiotic prophylaxis except for a closed reduction of fracture with internal fixation (ICD-9, 79.1), where cephtriaxone was used (as recommended by the Polish State Consultant for Microbiology), and for simpler operations such as an arthroscopy with no implant in place, where there was no antibiotic prophylaxis.

The basic demographics of patients and the characteristics of the SSIs have been collected. A retrospective analysis of the SSIs, based on the reports of lab-based monitoring, has been carried out in cooperation with the infection control team and doctors working at the unit or at the ambulatory care, in compliance with the ECDC definitions (https://ecdc.europa.eu/sites/portal/files/documents/HAI-Net-SSI-protocol-v2.2.pdf). The SSIs have been qualified as superficial/deep incisional or organ/space. The follow-up period was 30 days for the superficial SSIs and 90 days for deep or organ/space infections following arthroplasties.

Microbiological tests (wound swabs, abscess or biopsy aspirates) were drawn when ordered by the attending physician. Thirty-two percent of all materials had been collected through biopsies. Only the non-repeating isolates have been included in the study, excluding multiple strains coming from the same SSI case. The strains have been identified using BD Phoenix NID cards of the automated Phoenix 100 Becton Dickinson Diagnostic System (Becton Dickinson, Warsaw, Poland) according to the manufacturer’s instructions.

Antimicrobial susceptibility has been assessed according to the current guidelines of the European Committee on Antimicrobial Susceptibility Testing (EUCAST) (http://www.eucast.org). The results of drug sensitivity examinations have been interpreted in compliance with the EUCAST criteria. For *Acinetobacter baumannii*, ampicillin-sulbactam susceptibility was assessed according to the guidelines of the Clinical Laboratory Standards Institute (breakpoints *R* < 11, *I* = 12–15, *S* > 15). For the strains classified as multidrug-resistant (MDR), susceptibilities to colistin, tigecycline, ertapenem, imipenem, meropenem, amikacin, gentamycin, tobramycin, trimethoprim-sulfamethoxazole, ceftazidime and cefepime have also been examined.

Extended-spectrum beta-lactamase (ESBL) activity has been detected by means of a modified double-disk synergy test (DDST), using a combination of cefotaxime (5 μg), ceftazidime (10 μg), cefepime (30 μg) and aztreonam (30 μg) disks, placed 20 mm apart around a disk containing amoxicillin-clavulanate (20 μg/10 μg). Enhancement of the inhibition zone toward the amoxicillin-clavulanate disks was taken as a presumptive evidence of ESBL production.

The cumulative antibiogram reports have been prepared using the Formula for Rational Empiric Antimicrobial Therapy (FRAT) [13]. The latter had been calculated as the microorganism prevalence multiplied by the drug sensitivity rate—both figures based only on the microorganisms detected and antibiotic groups applied in the studied SSI cases.

Statistical measures have been calculated for the sample: the number of variants and the incidence for each variant, the arithmetic mean and the standard deviation.

The results have been analysed by means of PQStat ver. 1.6.0.428, using the chi-squared test or Fisher’s exact test. The Kendall rank correlation coefficient has been estimated for the changes in antibiotic groups and in other scales throughout the years. The *p* value was 0.05.

The use of the data has been approved by the Bioethical Committee of Sosnowiec Medical College (No. PW/WSM/36/17). All the data entered into the electronic database and analysed in the study had been anonymised.

## Results

Five thousand two hundred thirty-nine operations were performed at the ward in the studied period. On average, patients were aged 67 in the HPRO and KPRO, 49.6 in the OPRO and 49 in OMS. Male patients were operated on more frequently—their cases constituted 51.7–58.1% of prosthesis implantations and 70.2% of OMS.

An SSI has been detected 345 times, with an incidence rate of 6.6%. Post-operative in-hospital SSI case fatality rate was 0.0%. No correlation between the sex of the patient and the SSI incidence has been observed (chi^2^ = 0.86, df = 1, *p* = 0.353), but the age proved a significant risk factor (chi^2^ = 12.92, df = 5, *p* = 0.027): SSIs occurring most frequently for the patients of 46–59 and 60–75 years of age (Table 1).

**Table 1:** Characteristics of the patients operated at the Department and Clinic of Orthopaedic and Trauma Surgery in 2013–2015

	Studied patients		Incidence rate (%)	OR (95%CI)	*p* value
	With SSI *n* = 345	Without SSI *n* = 4894			
Age (year)					
0–15	4 (1.2)	172 (3.5)	0.07	0.3 (0.12–0.87)	*p* = 0.0272
16–29	59 (17.1)	784 (16.0)	1.13	1.1 (0.81–1.45)	
30–45	76 (22.0)	1074 (21.9)	1.45	1.0 (0.77–1.31)	
46–59	91 (26.4)	1185 (24.2)	1.74	1.1 (0.87–1.44)	
60–75	100 (29.0)	1287 (26.3)	1.91	1.1 (0.89–1.46)	
> 75	15 (4.3)	392 (8.00)	0.29	0.5 (0.31–0.88)	
Gender					
Female	139 (40.3)	2097 (42.8)	2.65	0.9 (0.72–1.12)	*p* = 0.3531
Male	206 (59.7)	2797 (57.2)	3.93		

*OR (95%CI)* odds ratio and 95% confidence interval, *SSI* surgical site infections

There has been a significant correlation between the type of the surgery and the clinical form of the SSI (Fisher’s exact test, *p* = 0.001): the latter was mostly superficial in arthroplasties (although there were also many deep incisional SSIs in the hip replacements), and deep incisional and organ/space in OMSs (Table 2). There has also been a significant correlation between the type of the surgery and the time to detect the SSI (Fisher’s exact test, *p* = 0.001). Thereby, the post-OMS SSIs were mostly diagnosed even during the first hospitalisation, whereas the post-arthroplasty SSIs were diagnosed most frequently at the ambulatory care. Re-hospitalisation was needed in 21.4% of KPROs and in 40.6% of HPROs (Table 2).

**Table 2:** Characteristics of patients, operations and SSIs

Risk factors	HPRO		KPRO		OPRO		OMS	
	SSI		SSI		SSI		SSI	
	No	Yes	No	Yes	No	Yes	No	Yes
	*n* = 552	*n* = 32	*n* = 2961	*n* = 14	*n* = 342	*n* = 18	*n* = 2961	*n* = 228
Duration of operation (min)								
Mean	140	155	110	120	60	65	120	125
SD	42	43	36	36	16	16	38	38
* p* value	0.0503		0.3123		0.1971		0.0556	
P75 (min)	160		130		94		135	
Age (years)								
Mean	67.0	69.1	66.8	69.0	49.8	46.7	47.9	62.0
SD	18	18	18	18	16	16	16	18
* p* value	0.5215		0.6564		0.4235		< 00001	
The duration of the pre-hospitalisation (days)								
Mean	6	6	6	6	4	4	3	4
SD	7	7	7	7	6	6	4	4
* p* value	1.0000		1.0000		1.0000		0.0003	
Gender (%)								
Male	55.3	53.1	58.1	57.1	51.5	55.6	70.6	64.5
Female	44.7	46.9	41.9	42.9	48.5	44.4	29.4	35.5
* p* value	0.8139		0.9413		0.7348		0.0520	
OR (95%CI)	0.8 (0.57–1.19)		0.07 (0.04–0.11)		0.7 (0.5–1.2)		1.1 (0.9–1.3)	
Cleanliness of the surgical site (%)								
Clean, clean-contaminated	99.8	100.0	99.6	100.0	99.7	100.0	93.0	82.0
Contaminated, dirty	0.2	0.0	0.4	0.0	0.3	0.0	7.0	18.0
* p* value	0.8096		0.8155		0.8183		< 0.0001	
OR (95%CI)	1.0 (0.61–1.66)		1.00 (0.48–2.11)		1.00 (0.51–1.96)		0.88 (0.72–1.1)	
State of the patient in the ASA scale (%)								
1 or 2	67.6	53.1	69.8	42.9	76.9	72.2	83.0	86.0
3 or 4 or 5	32.4	46.9	30.2	57.1	23.1	27.8	17.0	14.0
* p* value	0.0916		0.0349		0.6474		0.2503	
OR (95%CI)	0.8 (0.9–3.8)		3.0 (1.1–8.9)		1.3 (0.4–3.7)		0.8 (0.5–1.2)	
Incidence rate (%)	5.8		5.4		5.3		7.7	
Clinical form of the SSI, *n* (%)								
Superficial	16 (50.0)		12 (85.7)		15 (83.3)		97 (42.5)	
Deep	14 (43.8)		2 (14.3)		3 (16.7)		125 (54.8)	
Organ/space	2 (6.3)		0 (0.0)		0 (0.0)		6 (2.6)	
Time of detection of SSI, *n* (%)								
During hospital stay	7 (21.9)		4 (28.6)		4 (22.2)		139 (61.0)	
Post-disleftge	12 (37.5)		7 (50.0)		14 (77.8)		49 (21.5)	
Re-hospitalisation	13 (40.6)		3 (21.4)		0 (0.0)		40 (17.5)	

*ASA* the American Society of Anesthesiologists physical status classification, *HPRO* hip endoarthroplasty, *KPRO* knee endoarthroplasty, *OMS* other musculoskeletal, *OPRO* other prostheses, *OR (95%CI)* odds ratio and 95% confidence interval, *P75* 75th percentile of duration of operation, *SD* standard deviation, *SSI* surgical site infections

In the implantations—HPRO, KPRO, OPRO—the incidence rate amounted to 5.8%, 5.4% and 5.3%, respectively, and was not influenced by any of the risk factors we have been focusing on, except the overall condition of the patient measured in the ASA scale for the KPRO (chi^2^ = 4.45, df = 1, *p* = 0.0349) (Table 3).

**Table 3:** Most frequently isolated aetiological factors of SSIs at the Department and Clinic of Orthopaedic and Trauma Surgery in 2013–2015

Pathogen	*n* (%)	Ranking	MDR*, *n* (%)	Trend of occurrence
Gram-positive	225 (56.0)		n/a	
Coagulase-negative staphylococci	81 **(**20.1**)**	1		
*Staphylococcus aureus*	65 (16.1)	2	17 (26.2)**	Tau = 0.3; *p* = 0.60
*Enterococcus faecalis*	49 **(**12.2**)**	3	n/a	
*Enterococcus faecium*	10 **(**2.5**)**	9		
Others	20 **(**5.0**)**	8		
Gram-negative	175 (43.5)			
*Acinetobacter baumannii*	41 **(**10.2**)**	4	40 (97.6)	Tau = − 0.8; *p* = 0.20
*Escherichia coli*	30 **(**7.5**)**	5	4 (16.7)	Tau = 0.8; *p* = 0.20
*Klebsiella pneumoniae*	22 **(**5.5**)**	6	11 (50.0)	Tau = − 1.0; *p* = 0.12
*Enterobacter cloacae*	21 **(**5.2**)**	7	n/a	
*Proteus mirabilis*	20 **(**5.0**)**	8	7 (35.0)	Tau = 1.0; *p* = 0.12
*Pseudomonas aeruginosa*	20 **(**5.0**)**	8	4 (20.0)	Tau = 1.0; *p* = 0.12
Others	21 **(**5.2**)**	7	n/a	
*Candida* spp.	2 **(**0.5**)**			
Total	402 (100)		91 (22.6)	Tau = − 0.3; *p* = 0.60

*Other MDR 8 (3.8%)

**Only MRSA

*MDR* multidrug-resistant, including extended-spectrum beta-lactamases (ESBL) and methicillin-resistant *Staphylococcus aureus* (MRSA), *n/a* not available

The incidence rate was the highest in other musculoskeletal surgeries (OMSs), where it amounted to 7.7%. The rate was higher in the cases in which the patient had been being hospitalised for a long time prior to the surgery (*t* = − 3.64, df = 3187, *p* = 0.0003). The age of the patient (*t* = − 12.70, df = 3187, *p* < 0.0001) and the wound contamination class (chi^2^ = 35.66, df = 1, *p* < 0.0001) also influenced the incidence rate.

### SSI aetiology

Most of the isolated microbes were the Gram-positive cocci (56%, 225 strains, mostly coagulase-negative staphylococci (CoNS) and *Staphylococcus aureus*). Among the Gram-negative cocci, *Acinetobacter baumannii* was the most frequent (Table 3). The yeast-like fungi (0.5%) had been isolated from wound swabs.

MRSA prevalence was 26.2% (17 strains). The prevalence of MDR microorganisms was 22.6%, mostly in the Gram-negative bacilli: 97.6% of *Acinetobacter baumannii* and 50.0% of *Klebsiella pneumoniae* were multidrug-resistant. The share of MDR strains did not change during the studied period (tau = − 0.3, *p* = 0.60) (Table 4). On the basis of what the FRAT analysis has shown, the use of amikacin (only in combination therapy), imipenem and ciprofloxacin (in the absence of other therapeutic options) has been recommended as the most efficient in the empirical therapy of SSIs (Table 4).

**Table 4:** The cumulative antibiogram report, prepared in compliance with the Formula for Rational Empiric Antimicrobial Therapy (FRAT)

Pathogen	%*I*	Amikacin		Ceftazidime		Ciprofloxacin		Imipenem	
		%*S*	IF	%*S*	IF	%*S*	IF	%*S*	IF
Coagulase-negative staphylococcus	20.1	6	1.2	n/a	n/a	24	4.8	n/a	n/a
*Staphylococcus aureus*	16.2	50	8.1	n/a	n/a	53	7.0	n/a	n/a
*Acinetobacter baumannii*	10.2	3	0.3	0	0	4	0.4	17	1.7
*Escherichia coli*	6.0	82	4.9	71	4.3	65	4.0	100	6
*Klebsiella pneumoniae*	5.5	60	3.9	38	2.1	46	2.5	100	5.5
*Enterobacter cloacae*	5.2	95	4.9	71	3.7	87	4.5	100	5.2
*Proteus mirabilis*	5.0	67	3.4	68	3.4	50	2.5	29	1.5
*Pseudomonas aeruginosa*	5.0	100	5.0	89	4.5	68	3.4	80	4
Percentage of overall activity		43.3		24.6		29.8		32.7	

*n/a* not available; *%I* percentage frequency of isolation, *%S* percentage sensitivity of pathogen to antibiotic, *IF* antimicrobial impact factor: the likelihood that a pathogen would be sensitive to an antimicrobial drug, (%*I* × %*S*) ∕ 100

## Discussion

The ultimate goal of surveillance is to reduce the risk of nosocomial infections. The key means to achieve this are monitoring the infections (outcome measures) and overseeing the elements of hospitalisation (process measures), including certain procedures and hand hygiene. The data, including the information from a continuous prospective surveillance, should flow between the staff smoothly and quickly if the antibiotic policy of the hospital is to be effective.

However, rendering the surveillance satisfactorily effective is a difficult task. It is even more difficult in the countries where the hospital infection control is only now being introduced, such as Poland [14]. A targeted active surveillance in Poland is run reluctantly and by few hospitals, and the incidence is much higher than expected [15, 16]. Other countries of the region have been facing similar problems, e.g. in 2000, the SSI incidence rate for different types of surgeries in Russia was 9.5% [17].

A rational infection control should include laboratory-based monitoring. Microbiological diagnostic research is a key element of identification and treatment of HAIs, SSIs included [18]. The laboratory-based surveillance at the studied hospital is run mostly by diagnostician’s microbiologist. The epidemiological figures show that the cooperation between them and the personnel of the unit—that is the detection and identification of the SSIs, and acquiring the materials for clinical research—was very good. However, the SSI incidence is alarming high. This is probably due to insufficient compliance of infection prevention and control practices—e.g. antibiotic prophylaxis, surgical hand preparation, aseptic non-touch technique and wound care—resulting from the lack of a continuous presence of an infection control professional (ICP) at the ward and the lack of checking compliance of such procedures. Thereby, a continuous laboratory-based monitoring has not replaced the epidemiological surveillance successfully, from the prevention point of view.

The highest incidence has been observed in OMSs. The rate used to be much lower in the past: in 2003–2004, it amounted to 1.2–1.3% in Poland [19, 20] and to 0.63% (patients with no risk factors) or 1.78% (patients with numerous risk factors) in the USA [5]. What is more, the SSI rate for arthroplasties at the studied ward is too high—several times higher, while compared with the figures from other Polish units, or from the USA [6, 8, 19–22]. This said, the analysis of yearly (2011–2014) SSI incidence rates in EU countries has shown a decreasing trend in the KPROs (*p* < 0.001).

It is difficult to identify a single cause of such an elevated SSI incidence just on the basis of this laboratory-based study; it is vital that a systematic and active surveillance, overseen by an ICP, be introduced directly at the ward. It should not only identify and register but also prevent the infections [23–26].

In Poland, SSI surveillance is rarely maintained once the patient has left the hospital [27, 28]. At the studied hospital, however, an effective laboratory-based monitoring has been implemented in the ambulatory care. Many of the SSIs were detected post-discharge, with no re-hospitalisation. A Finnish study [29] has also observed an effective post-discharge surveillance, but only some 2/3 of the SSIs detected by it were confirmed in the microbiological analysis. This may be due to the short post-surgery hospitalisation, which lasted 8 days in the Finnish study [29] (or 12 days in China [30]), and was longer in ours. We believe that the high SSI incidence is directly connected to an effective post-discharge surveillance, rare in other European countries.

An elevated number of deep incisional infections had been expected, as already described by other authors [8, 13]. A Swiss research, however, points to a lower share of deep incisional infections: 28.4% for hip replacements and 44.8% for knee replacements, morbidity amounting to 1.4% and 0.9% respectively [31].

Most of the aetiological factors of the SSIs are Gram-positive cocci, which are typical for orthopaedic patients [32, 33], so is the fact that the coagulase-negative staphylococci (CoNS) constitute 20% of the factors [34]. On the other hand, CoNS (possible skin contaminants) can be the evidence of excessive sensitivity in detecting infections or finding the infections. What is concerning is the frequent presence of *Acinetobacter baumannii*, much higher than in the ECDC data on orthopaedic infections, where it is estimated at 0.7% [34]. *A. baumannii* has also proved a major aetiological problem at the ICU of the studied hospital, where it constituted 13.5% of all pathogens from different clinical samples. 76.5% of strains were extensively drug-resistant [35].

The SSIs caused by non-fermentative bacilli *Acinetobacter baumannii* and *Pseudomonas aeruginosa* are particularly difficult to treat, as these bacilli are often multidrug-resistant [36–38].

This retrospective study had some limitations. Firstly, the research involves only one centre. Secondly, in the period studied, despite participation in the multiprofile programme, the infection registration method was not validated; hence, its sensitivity is not known in this particular case. Furthermore, the other post-operative complications were not analysed, which can extend the hospital stay, delay rehabilitation and impact patient satisfaction like non-surgical infections, non-union and delayed union, or post-operative nausea and vomiting [39–42]. Also, demographic information for the study population was limited; thus, data on the characteristics of the patients (for example, peri-operative bacteriuria, bacterial contamination rate of electrocautery tips, smoking) were unavailable, as well as differences in the type of care received by the patients, for example, dressing type [43–45].

To conclude, the study has validated the usefulness of a laboratory-based monitoring of SSIs. On the other hand, it has revealed that the infection control was a significant problem at the studied orthopaedic unit: the SSI rates were significantly higher than expected. This phenomenon may be due to inadequate local infection control. We suggest implementing the infection control and prevention based on evidence-based medicine [23]. An active unit-based surveillance—not only monitoring—is even more important. Such an active SSI surveillance should be adopted by other hospitals in Poland.

A cumulative unit-based antibiogram reflects the drug-susceptibility pattern for the strains from the infections acquired at the unit. It may be useful in choosing the empirical antibacterial therapy.

## Data Availability

The datasets generated or analysed during this study are available and can be accessed from Grzegorz Ziolkowski (e-mail: nc3@wp.pl) on reasonable enquiry.
